# 91 Comparison of a Polylactic Acid Skin Substitute to Porcine Xenograft for Pediatric Partial Thickness Burns

**DOI:** 10.1093/jbcr/irac012.094

**Published:** 2022-03-23

**Authors:** Kaylyn B Pogson, Lori Chrisco, Rabia Nizamani, Felicia Williams, Booker King

**Affiliations:** University of North Carolina at Chapel Hill School of Medicine, Chapel Hill, North Carolina; North Carolina Jaycee Burn Center, Chapel Hill, North Carolina; UNC Jaycee Burn Center, Chapel Hill, North Carolina; UNC Jaycee Burn Center, Chapel Hill, North Carolina; UNC Jaycee Burn Center, Chapel Hill, North Carolina

## Abstract

**Introduction:**

In August of 2020, our institution transitioned from porcine xenograft to a polylactic acid skin substitute for the management of pediatric partial thickness burns. This change in treatment was due to the discontinuation of porcine xenograft by the primary supplier to the United States. We sought to make a length of stay (LOS), postoperative pain score, postoperative dressing change, and cost analysis of the polylactic acid skin substitute as compared to porcine xenograft for the treatment of pediatric burns.

**Methods:**

Patients were identified using an institutional Burn Center registry and linked to clinical and administrative data. All pediatric patients admitted between January 1^st^, 2019 and March 31^st^, 2021 who sustained partial thickness burns were eligible for inclusion. LOS, burn etiology, total burn surface area (TBSA), postoperative pain scores, postoperative dressing changes, complications, infections, and hospital cost were evaluated.

**Results:**

A total of 259 patients were identified, 47 of whom received the polylactic acid skin substitute and 212 of whom received xenograft. Average age for polylactic acid skin substitute patients was 5.4 years with 51.1% male, average age for xenograft patients was 4.6 years with 58.5% male. Average LOS for polylactic acid skin substitute patients was 3.4 days and 3.2 days for xenograft patients (p = 0.45). Etiology of burns was 83.0% scald and 10.6% flame for polylactic acid skin substitute patients and 80.2% scald and 9.40% flame for xenograft patients (p = 0.66 and p = 0.71, respectively). Polylactic acid skin substitute patients had an average TBSA of 5.3% and xenograft patients an average TBSA of 4.3% (p = 0.11). Postoperative pain scores on postoperative day (POD) 1 were 1.1 for polylactic acid skin substitute and 1.2 for xenograft (p = 0.13). Average number of inpatient postoperative dressing changes was equivalent between the polylactic acid skin substitute and xenograft (p = 0.62), while average day of first postoperative dressing change was POD 10.9 for the polylactic acid skin substitute and POD 9.9 for xenograft (p = 0.15). Neither group had postoperative infections, though xenograft had a complication rate of 1% with 2 patients while the polylactic acid skin substitute had 0%. Polylactic acid skin substitute patients had an average hospital cost of $28,415 and xenograft patients an average of $27,935 (p = 0.80).

**Conclusions:**

A polylactic acid skin substitute is equivalent to porcine xenograft in LOS, postoperative pain, postoperative dressing changes, and cost in the setting of similar age, burn etiology, and %TBSA. More analysis with wound healing indices and safety profiles could determine the clinically superior choice.

